# Establishment and Clinical Application of an Artificial Intelligence Diagnostic Platform for Identifying Rectal Cancer Tumor Budding

**DOI:** 10.3389/fonc.2021.626626

**Published:** 2021-03-08

**Authors:** Shanglong Liu, Yuejuan Zhang, Yiheng Ju, Ying Li, Xiaoning Kang, Xiaojuan Yang, Tianye Niu, Xiaoming Xing, Yun Lu

**Affiliations:** ^1^ Department of Gastrointestinal Surgery, The Affiliated Hospital of Qingdao University, Qingdao, China; ^2^ Department of Pathology, The Affiliated Hospital of Qingdao University, Qingdao, China; ^3^ Department of Blood Transfusion, The Affiliated Hospital of Qingdao University, Qingdao, China; ^4^ Department of Operating Room, The Affiliated Hospital of Qingdao University, Qingdao, China; ^5^ Nuclear & Radiological Engineering and Medical Physics Programs, Woodruff School of Mechanical Engineering, Georgia Institute of Technology, Atlanta, GA, United States

**Keywords:** rectal cancer, tumor budding, artificial intelligence, pathological diagnosis, Faster R-CNN

## Abstract

Tumor budding is considered a sign of cancer cell activity and the first step of tumor metastasis. This study aimed to establish an automatic diagnostic platform for rectal cancer budding pathology by training a Faster region-based convolutional neural network (F-R-CNN) on the pathological images of rectal cancer budding. Postoperative pathological section images of 236 patients with rectal cancer from the Affiliated Hospital of Qingdao University, China, taken from January 2015 to January 2017 were used in the analysis. The tumor site was labeled in Label image software. The images of the learning set were trained using Faster R-CNN to establish an automatic diagnostic platform for tumor budding pathology analysis. The images of the test set were used to verify the learning outcome. The diagnostic platform was evaluated through the receiver operating characteristic (ROC) curve. Through training on pathological images of tumor budding, an automatic diagnostic platform for rectal cancer budding pathology was preliminarily established. The precision–recall curves were generated for the precision and recall of the nodule category in the training set. The area under the curve = 0.7414, which indicated that the training of Faster R-CNN was effective. The validation in the validation set yielded an area under the ROC curve of 0.88, indicating that the established artificial intelligence platform performed well at the pathological diagnosis of tumor budding. The established Faster R-CNN deep neural network platform for the pathological diagnosis of rectal cancer tumor budding can help pathologists make more efficient and accurate pathological diagnoses.

## Introduction

Rectal cancer is one of the most common gastrointestinal malignancies, with rising morbidity and mortality. Ranking third and the fourth among malignancies in terms of incidence and mortality, respectively, it has a great impact on life expectancy and quality of life (1). The 5-year survival rates of early-stage (stages I and II) patients are 93.8 and 77.4% respectively, but those of late-stage (stages III and IV) patients are 14 and 7%. Therefore, for the development of new clinical intervention programs, it will be clinically significant to find the key clinical and molecular pathological features that affect patients’ survival and prognosis (2).

The tumor, node, metastasis (TNM) staging model has long been used as the standard for gastrointestinal tumors and has been recognized as a reliable indicator of a patient’s prognosis. In recent years, it was found that tumor budding has important diagnostic value in the clinicopathology and prognosis of gastrointestinal tumors (3, 4). The incidence of tumor budding in rectal cancer cases with lymph node metastasis and lymphatic invasion is significantly higher than in those without lymph node metastasis, while local recurrence and liver metastasis are also more common in those with tumor budding (5). The incidences of tumor budding and lymph node metastasis among rectal cancer patients are closely related to TNM stage. Tumor budding is an independent risk factor for rectal cancer invasion and prognosis (6).

Tumor buds are small, focal tumor cell populations consisting of undifferentiated individual tumor cells or four cells scattered in the anterior margin of infiltrating tumor. It is not a static histological feature but a dynamic process of aggressive tumor progression with infiltrative and metastatic potential, and it is not just simple detachment of tumor cells but an important step in the malignancy from focal to systemic development (7). Tumor budding is considered a sign of cancer cell activity and the first step of tumor metastasis. It is microscopically identified by pathologists through hematoxylin and eosin (HE) staining. Histologically, tumor budding is more common in higher grade adenocarcinomas with poorer differentiation, where individual or small focal tumor cell groups are separated from the tumorous gland in the anterior margin of the tumor infiltration and enter the interstitium as fine lines or small striations (8). Cancer cells and nuclei with tumor budding are heterotypic, irregular in shape, abundant in cytoplasm, eosinophilic, often fused, and heavily stained in the nucleus (9). However, since the foci of budding tumors are rather small and sometimes contain a single isolated tumor cell, or with a severe inflammatory reaction around the budding tumor cells, it is difficult to observe and identify tumor budding on HE staining. This is one of the reasons for the low budding rate reported by pathologists in rectal tumors reported. Especially in China, the large population and large amounts of pathologic information, coupled with a severe shortage of pathologists, have led to high workload and pressure on pathologists in hospitals of different regions and at varying levels of diagnosis, often causing different pathologists to reach vastly different diagnoses from the same pathology data.

Artificial intelligence (AI) boasts the stable error, fast computing speed, and high accuracy of computers in processing data (10). By using deep learning techniques to train an AI on the tumor budding characteristics from a large number of HE-stained rectal cancer sections and their corresponding diagnostic results, such an AI would be able to read pathology images and correctly diagnose tumor budding as well as pathologists do (11). Currently, AI technology can partially ease the burden of clinicians in diagnosing and identifying skin cancer, reading and interpreting breast lymph node metastases, etc. The AI-assisted reading and interpretation of pathological images can improve the reading speed and reduce human-induced errors while enhancing the overall accuracy of the pathological diagnosis (12).

To improve the accuracy of postoperative pathological diagnosis of rectal cancer patients and reduce the workload of pathologists, we established an automated diagnostic system for rectal cancer tumor budding by training an AI on a large number of images from HE-stained pathological sections with rectal cancer tumor budding through the deep learning technique. We then assessed the accuracy of this automated diagnostic system on a validation dataset to verify its clinical feasibility. Here, we report the system-building process and learning outcome.

## Materials and Methods

### General Information

In this retrospective clinical study, we included 236 rectal cancer patients received by the Affiliated Hospital of Qingdao University from January 2015 to January 2017. The inclusion criteria were as follows: the patient was pathologically diagnosed with rectal cancer through preoperative colonoscopic biopsy, had no malignant tumors in other parts of the body, underwent radical resection of rectal cancer, and had a complete postoperative pathological diagnostic result. The exclusion criteria were as follows: the patient had preoperationally or postoperationally undetermined rectal space–occupying lesions, underwent no surgical treatment or radical resection, or had a rectal malignancy of other sources. The protocols used in the study were approved by the Ethics Committee of the Affiliated Hospital of Qingdao University and conducted in full accordance with ethical principles. Specifically written informed consent was obtained from the patients that we could use their data without revealing their privacy.

### Establishment of a Tumor Budding Database From Rectal Cancer Pathology Sections

The age, sex, tumor site and size, and lymph node metastasis were collected from the patient’s clinical history and pathology report. The patient’s pathological sections were reviewed by pathologists with gastrointestinal pathology expertise to assess pathological features related to tumor budding. The process of evaluating tumor budding was as follows. Rectal cancer tissues were immersed in 4% paraformaldehyde for 4h, and transferred to 70% ethanol. Individual rectal cancer tissues were placed in processing cassettes, dehydrated through a serial alcohol gradient, and embedded in paraffin wax blocks. 5-um-thick tissue sections were dewaxed in xylene, and washed in PBS. And then stained with hematoxylin and eosin (H&E). After staining, sections were dehydrated through increasing concentration of ethanol and xylene. All HE-stained sections were microscopically examined at low magnification (×100), in which the area with the highest budding density was searched in the anterior of the tumor invasion and the total number of buddings was counted in 10 view fields of the area at high magnification (×400). If no obvious budding area was found at low magnification, then 10 view fields at high magnification were randomly selected, and the buddings were counted. The criteria for judging tumor budding were as follows: solitary single cancer cells or cell clusters consisting of less than five cancer cells in the front of tumor invasion area; no tumor budding was judged if there was no single or cancer cell clusters in the front of tumor invasion area (13). All these images were based on digital histopathology slides. The whole images were taken with 3DHISTECH scanner-Pannoramic SCAN 150. The Pannoramic SCAN 150 offers world class bright-field whole slide scanning on up to nine channels. An outstanding 0.12 μm/pixel (in Bright-field) resolution is achieved with the 40×/NA 0.95 (equivalent to 80×magnification in BF) Carl Zeiss Plan-Apochromat objective. The slides were scanned with 40x objective. A total of 2,801 images of HE-stained postoperative pathological sections from 236 patients were set as the rectal cancer tumor budding database for the establishment and verification of the deep learning platform. When grouping the dataset, we first shuffled the numbered inclusion data and then randomly chose 80% of the data as the training set and the remaining 20% of the data as the verification set. To train the artificial intelligent diagnosis system, the tumor budding was marked independently in each image by two senior experienced pathologists with more than 5 years of working experience (Zhang YJ and Xing XM), and differences were resolved by consensus.

### Training Process of the Automatic Diagnostic Platform for Rectal Cancer Tumor Budding

(1) We used 80% of the randomized data as the training set for the AI platform, in which the patient’s full-sequence images were imported to the neural network system, with the labeled images as positive ones and the unlabeled images as the negative controls. The image was scaled to 512×557 pixels in size and normalized so the pixels of each channel had a standard normal distribution with a mean of 0 and a variance of 1. To speed up the learning, the Faster region-based convolutional neural network (R-CNN) algorithm was used for training. In it, the ResNet101 and the stochastic gradient descent (SGD) optimizer were used to extract image features, and the target detection model was individually trained at each image level.

(2) Automatic recognition of rectal cancer tumor budding based on deep learning. The standard Faster R-CNN architecture mainly includes a feature extraction network, a region production network (RPN), and a Fast R-CNN target detection network. Of these, the feature extraction network used the VGG16 network model to abstract the image features of rectal cancer tumor budding and generate the convolutional feature map; the RPN performed a sliding scan of all features present in the convolutional feature map, and at each sliding window position, multiple candidate regions were simultaneously chosen. Assuming the maximum number of possible proposals for each position is *k*, then the regression layer had 4*k* outputs and encoded the coordinates of *k* bounding boxes. The classification layer outputted 2*k* scores and estimated the probability at which each proposal was a target. Relative to the *k* reference bounding boxes that are called anchor points, the *k* proposals were parameterized. Each of the anchor points was at the center of the sliding window at issue and was associated with a scale and aspect ratio (14, 15). By default, three scales and three aspect ratios were used, giving rise to *k* = 9 anchor points at each sliding position.

To obtain the candidate region, each anchor point was assigned a binary category label (i.e., a target or not a target). A positive label was assigned to two types of anchor point: (1) any anchor point that overlapped with the ground-truth bound box at the highest intersection-over-union (IoU), and (2) any anchor point that overlapped with the ground-truth bound box at an IoU of over 0.7. For all ground-truth bound boxes, if the IoU ratio of an anchor point was lower than 0.3, a negative label was assigned to the nonpositive anchor point. In this way, the possible rectal cancer tumor budding region was generated on the convolutional feature map, and then the adjacent regions were merged using the nonmaximum suppression method to reduce candidate regions for training and thus avoid a large amount of unnecessary repeated computation in the subsequent target detection and classification. The RPN and the Fast R-CNN shared the convolutional feature map, and after passing through the pooling layer of the region of interest and two sub-fully connected layers, the coordinates of the predicted bound box and the probability of the category were obtained. In the test, the RPN and the Fast R-CNN were performed with alternating two-stage training, the parameters were continuously fine-tuned in the iterations, and the position of candidate bound box was calibrated through bounding box regression to obtain the final optimal result (16).

(3) The establishment of the AI system for assisted diagnosis of rectal cancer tumor budding in this study included two processes: training and recognition. The four-step Fast R-CNN iterative training process was as follows: (1) An image of the labeled rectal cancer budding sequence was inputted to the Fast R-CNN network, the convolution feature map was outputted through the initial convolution feature extraction layer, and the parameters of the RPN were adjusted using the feature map and the label information, completing one training session for the RPN and the feature vector of the region of interest. (2) The same images were inputted to the Fast R-CNN network, the convolution feature map was outputted through the initial convolution feature extraction layer, and the feature region was produced by inputting the region of the convolutional feature map that had completed the first training session. The output was obtained by inputting the feature vector of the region of interest together with the convolutional feature map, and the training was then performed on the Fast R-CNN network through back-propagation. (3) The learning rates of Fast R-CNN and all convolutional layers sharing the RPN were set to 0. The convolutional layer unique to the RPN was retrained by inputting identical images and employing the Fast R-CNN that had completed the first training session. (4) The learning rates of Fast R-CNN and all convolutional layers sharing the RPN were set to 0, and the convolutional layer unique to the Fast R-CNN was retrained by inputting identical images.

(4) To verify the automatic diagnostic platform for rectal cancer tumor budding, the postoperative pathological data of 100 rectal cancer patients were collected from multiple centers and were used in the clinical test and verification of the established AI-assisted platform for the pathological diagnosis of rectal cancer tumor budding. The postoperative pathological data of rectal cancer patients were collected from multiple centers, and the final diagnostic conclusion on each case was made through rigorous analyses by a number of pathologists from different centers. The patients’ images, used as the verification dataset in this study, were inputted to the automatic diagnostic platform. The delineation of the tumor budding area by the platform was compared with the result of the postoperative pathology report, and the cases correctly identified by the platform were counted. The learning outcome of the AI platform was evaluated through the area under the receiver operating characteristic (ROC) curve (AUC).

### Statistical Methods

The measurement and count data were analyzed using the SPSS20.0 statistical software. The machine learning outcome was analyzed using the Python 3.7 programming language, and the Classification-report program of the Metric Module was used to generate the multiclassification report. T. The numbers of true positives and false positives on the nodule were counted, based on which the true-positive rate and false-positive rate under different probability thresholds were calculated. The ROC curve was plotted and its micro-AUC calculated.

## Results

**Figure 1:**
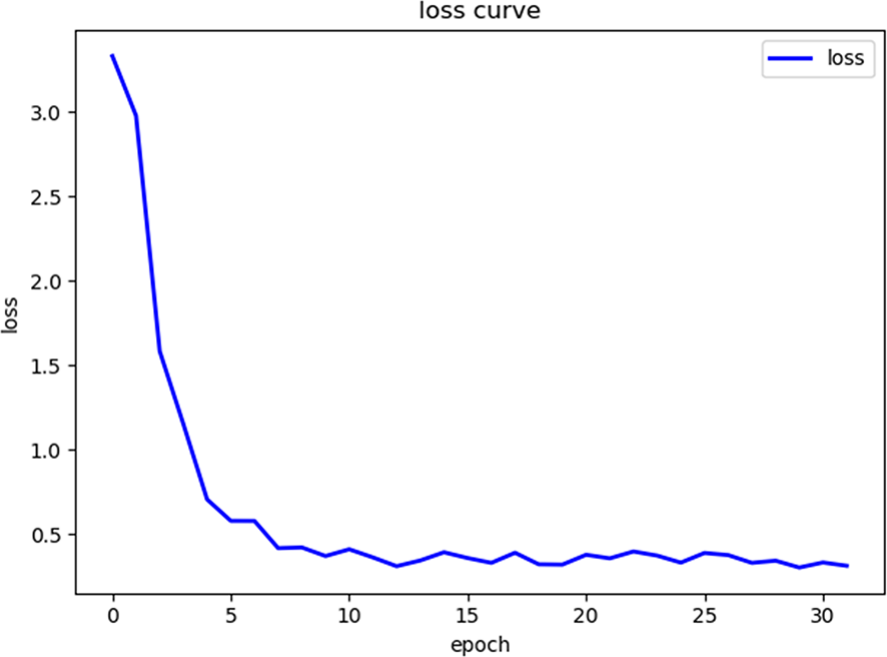
The loss curve of the machine learning process shows that as the number of repetitions of the machine learning data increases, the loss function value gradually decreases.

**Figure 2:**
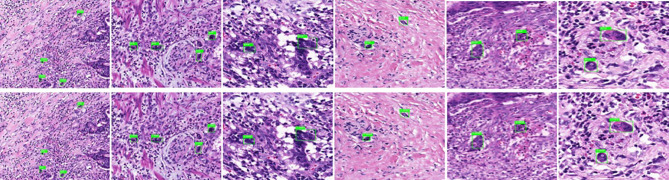
Annotated tumor budding by computer and pathologists in training group. The upper panels show labeling by the pathologists, and the lower panels show labeling by computers.

**Figure 3:**
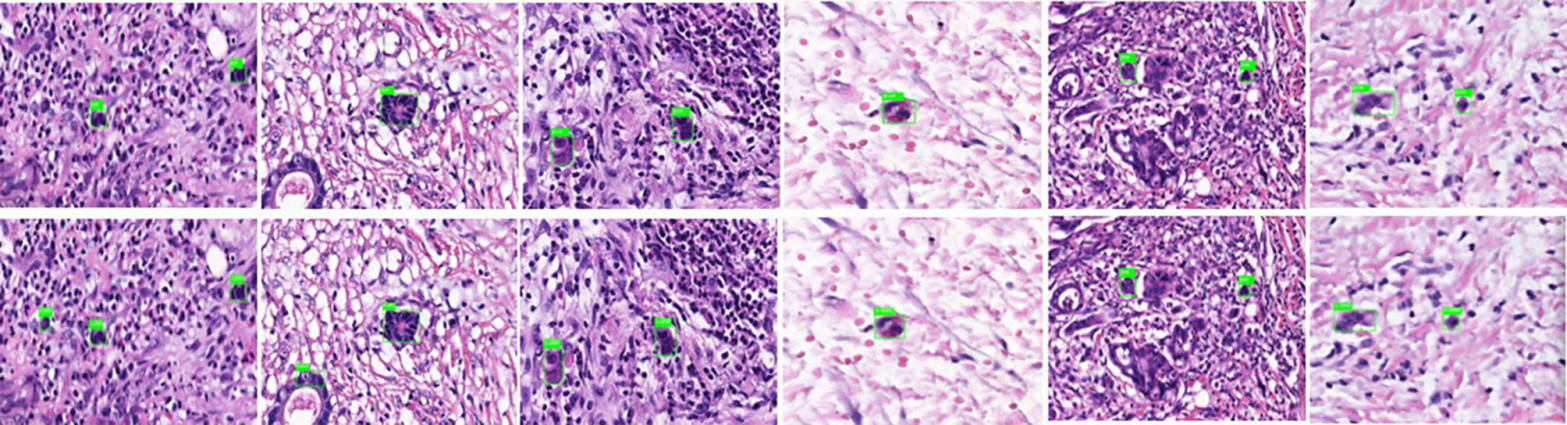
Annotated tumor budding by computer and pathologists in test group. The upper panels show labeling by the pathologists, and the lower panels show labeling by computers.

**Figure 4:**
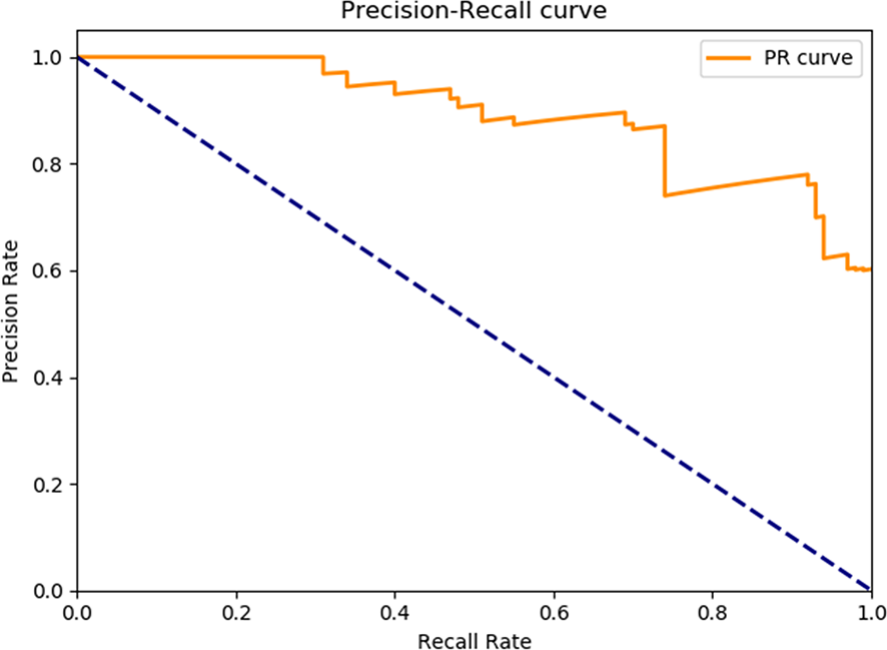
The precision-recall curve of the Faster R-CNN training process. The mAP of the main collection is the average of the average precisions from all classes. When the mAP value is closer to 100%, Faster R-CNN identification is more precise. mAP, Mean average precision; R-CNN, Region-based convolutional neural network.

**Figure 5:**
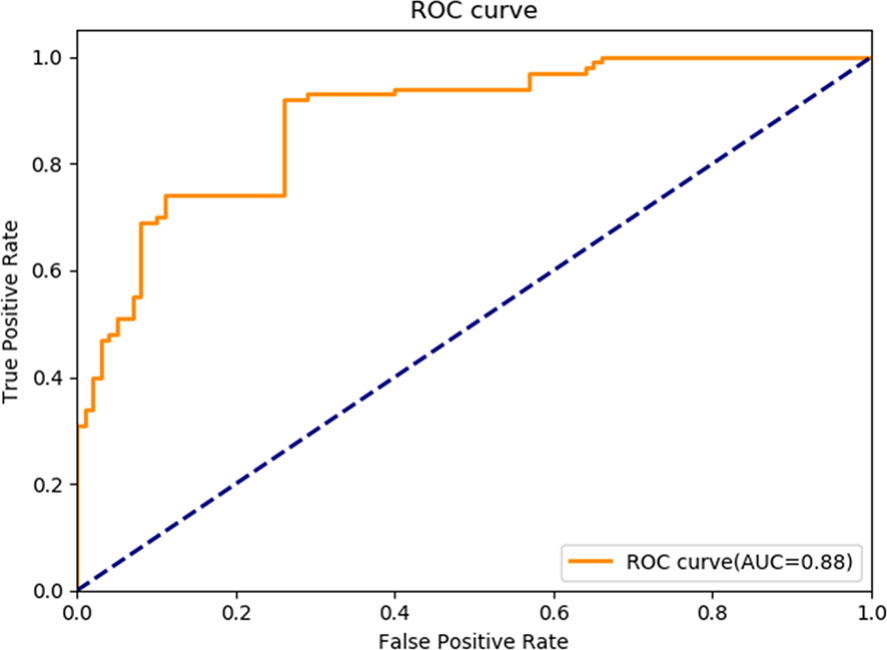
Receiver operating characteristic curve of Faster R-CNN in diagnosing tumor budding. AUC, Area under the receiver operating characteristic curve; R-CNN, Region-based convolutional neural network; ROC: Receiver operating characteristic.

1. General information of the patients and rectal cancer tumor budding database: A total of 236 patients with rectal cancer (153 males and 83females) were included. Among them, according to preoperative colonoscopy and magnetic resonance imaging reports, 69 had lower rectal cancer, 121 had middle rectal cancer, and 46 had upper rectal cancer.
2. In the training process, 80% of the randomized data were used for platform training. The VGG16, which contains 13 convolutional layers and three fully connected layers and has been pretrained in ImageNet, was used for the initialization of the feature extraction network. All the weights of the RPN and the feature vector Fast R-CNN of the region of interest were assigned with random values ​assuming a zero-mean Gaussian distribution with a standard deviation of 100. A two-stage training process was adopted, which included 80,000 iterations of RPN candidate region training and 40,000 iterations of classification and regression of the feature vector based on the candidate region (momentum: 0.9; weight decay: 0.0005; scale of anchor: 1282, 2562, 5122; aspect ratio of anchor: 0.5, 1, 2). In the training process, the deep learning network parameters (e.g., weight) were adjusted based on the data of end-to-end back-propagation that were obtained using the SGD method to decrease the loss function value and make the network converge. As shown in **Figure 1**, the attenuation function numerical curve of the Faster R-CNN network indicates that after training, Faster R-CNN achieved good attenuation for the identification and diagnosis of rectal cancer tumor budding.
3. In the identification experiment of this study, 20% of the randomized data were used as the verification set, and the experimental data were inputted to the trained Faster R-CNN model. Then the convolutional feature map was generated using the feature extraction network and screened through the RPN to produce putative tumor budding regions. Lastly, the site and probability of the tumor budding region were outputted through regression and classification on the convolutional feature map and the produced region using the feature vector of the region of interest, and the recognition result is shown in **Figure 2** (upper panels: labeling result by pathologists; lower panels: labeling result by the computer).
4. To evaluate the learning effectiveness of the Faster R-CNN deep neural network, 562 tumor budding sequence images were randomly sampled from the established database for training to be used as the test dataset and inputted to the trained Faster R-CNN. The identification results of test group are shown in **Figure 3** (upper panels: labeling result by pathologists; lower panels: labeling result by the computer). To understand the machine learning process, the precision and recall rates of the nodule category in the training dataset were recorded to plot the precision–recall (PR) curve, and the AUC was 0.7664, i.e., average precision (AP) = 0.7664. Because the experiment only had one category (i.e., nodule), mean AP = AP = 0.7664, indicating that the training of Faster R-CNN was good. Thus, for rectal cancer tumor budding sequence images, the training of Faster R-CNN was effective (**Figure 3**).
5. The clinical test and verification of the established AI platform were conducted using the postoperative pathological data of 100 cases of rectal cancer collected from multiple centers. The final diagnostic conclusion was obtained by rigorous analysis by multiple pathology experts from different centers. The processed image data were inputted to the trained Faster R-CNN to obtain the diagnostic result by the AI system, which was then compared with that by the experts, based on which the sensitivity, specificity, and accuracy of the AI diagnostic system for tumor budding were analyzed. To more directly examine and analyze the detection and classification results of the AI system, the labeled region of the test dataset was submitted to true positive/false positive classification, based on which the true-positive rate and false-positive rate under different probability thresholds were obtained, and the ROC curve was plotted (**Figure 4**). The AUC, which reflects the accuracy of the labeling of the data, was 0.88. Based on the ROC shown in **Figure 5**, the AUC of the Faster R-CNN–based AI assisted diagnosis, calculated using the trapezoidal method, was 0.88, indicating that the AI-assisted diagnosis was very effective. In the test, the automatic identification time of the Faster R-CNN–based AI diagnosis system for tumor budding images was 22 s for each patient, which was much shorter than the time taken by pathologists (5–8 min on average).

## Discussion

In this study, we established an AI-assisted diagnostic platform for assisted diagnosis of rectal cancer tumor budding using a Faster R-CNN–deep neural network algorithm to help pathologists make more accurate diagnoses, which is clinically significant for the assessment of the prognosis of rectal cancer patients and the choice of treatment. We collected and labeled a total of 2,801 images of HE-stained postoperative pathological sections from 236 rectal cancer patients and established a rectal carcinoma tumor budding pathology database based on the pathological result. Based on the database, we established an AI-assisted platform for automatic identification of rectal cancer tumor budding through Faster R-CNN, which was verified in the test dataset as well as through postoperative pathological clinical data that were collected from multiple centers. The verification results showed that the area under the ROC curve of the AI-assisted platform for the diagnosis of rectal cancer tumor budding established in this study was 0.88, with a high accuracy and a diagnosis time of 22 s/patient, much shorter than the time taken by pathologists, indicating that the system has good clinical feasibility and is more accurate and efficient than the traditional diagnostic method.

The combination of AI and medicine has greatly improved the efficiency and accuracy of medical treatment. Deep learning algorithms have been successfully applied in the diagnosis of skin cancer, prostate cancer, and breast cancer (17). The AI technology has also been used to make experimental diagnosis of metastatic lymph nodes in MR images of rectal cancer patients and obtained satisfactory experimental results (18, 19). In this study, we used the more sophisticated Faster R-CNN algorithm to enable the accelerated detection and learning of the target, thereby greatly reducing the time for training and recognition. The Faster R-CNN algorithm uses the image feature structure in ImageNet as the feature extraction network and the VGG16 model to initialize the network. ImageNet contains the data of 10 millions of images and object tags, and VGG16 is a very mature ImageNet-based deep neural network model for image feature extraction and classification, based on which the migration training on the feature extraction network was performed in this study, so the feature extraction training was based on the ImageNet big data, which enabled high-precision feature extraction from a relatively small amount of new data to achieve high-precision recognition (20, 21). Compared with improving the accuracy of machine imaging and the diagnostic capability of pathologists, training AI can make longer strides in a shorter time (22, 23). In terms of image processing, we adopted image standardization to generate more uniform input data, which improved the convergence speed and accuracy of the data processing while enabling the processing of data from different centers.

This study was a retrospective analysis of single-center data, which is a limitation. To further verify the clinical application value of Faster R-CNN in the AI-assisted diagnosis of rectal cancer tumor budding, we will conduct a prospective study on multicenter clinical data to examine the potential of Faster R-CNN–based AI-assisted diagnostic and prognostic assessment of rectal cancer tumor budding. Moreover, other tissue staining of pathological sections such as immunohistochemistry for tumor budding diagnosis and more human-machine confrontation test are needed to increase the credibility of system.

To solve various problems in the pathological diagnosis of rectal cancer tumor budding, e.g., relying on the pathologist’s subjective judgment, the poor objectivity and reproducibility, we established an AI-assisted diagnostic platform for the assisted diagnosis of rectal cancer tumor budding using Faster R-CNN with a deep neural network to help pathologists make more accurate diagnoses. Advancements in this technology will have great clinical value for the pathological staging of rectal cancer and the choice of patient treatment regimens.

## Data Availability Statement

The datasets presented in this study can be found in online repositories. The names of the repository/repositories and accession number(s) can be found in the article/supplementary material.

## Ethics Statement

The studies involving human participants were reviewed and approved by Medical Ethics Committee of Affiliated Hospital of Qingdao University. The patients/participants provided their written informed consent to participate in this study.

## Author Contributions

All authors listed have made a substantial, direct, and intellectual contribution to the work and approved it for publication.

## Funding

The study was supported by the National Natural Science Foundation of China (Grant No. 81802888) and the Key Research and Development Project of Shandong Province (Grant No. 2018GSF118206; No. 2018GSF118088).

## Conflict of Interest

The authors declare that the research was conducted in the absence of any commercial or financial relationships that could be construed as a potential conflict of interest.
